# The 10th anniversary: Journal of Intensive Care

**DOI:** 10.1186/s40560-024-00743-1

**Published:** 2024-08-07

**Authors:** Hiroshi Morisaki

**Affiliations:** Keio University, Tokyo, Japan

The *Journal of Intensive Care* (JINC), launched on October 23, 2013 [1], has consistently been ranked within quartile1 (Q1) in the category of Critical Care Medicine, according to the 2024 Journal Citation Reports^™^. First, I would like to express my sincere appreciation and congratulations to all those who have submitted, reviewed, and read the manuscript, as well as those who have supported this journal over the past decade.

Reflecting on the past, JINC was established as an official journal of the Japanese Society of Intensive Care Medicine (JSICM), which had been publishing its official journal (primarily in Japanese or English) since its establishment in 1994. Considering the presence of several top-ranked and leading journals in our area of expertise, I initially questioned whether there was room for a new journal amid significant economic, time, and other expenses. However, JSICM wisely selected Professor Satoshi Gando as the founding Editor-in-Chief (EIC), who promptly and effectively defined the scope, determined the publisher, and addressed other essential issues, including the open-access publishing model and public relations at the pre-editorial committee. Through such enthusiastic and wide-ranging efforts under his outstanding vision, the number of new submissions gradually but steadily increased: from 146 new manuscripts in 2014 to 341 in 2017 [2], with over 75% coming from other continents.

I assumed the role of the EIC at the beginning of 2018 [2] and reorganized the international and domestic members of the Editorial Board to meet essential requirements including regular and prompt publications, consistent editorial policy with precise review, internationality and diversity, and appropriate citation achievements. Due to various editorial and other efforts during 2018, the geographical distribution of our journal’s website visitors became more widespread and international: 31% of users were from the United States, whereas only 5% were from Japan. Following valuable advice from the Clarivate Analytics Japan office, I sent a formal letter in April 2019, accompanied by JINC’s achievements, requesting their careful consideration to grant an official impact factor. Contrary to our modest expectations, we received a prompt and promising reply, indicating that “Upon investigation into the issue you required, we have determined the need to escalate this case to our Publisher Relations Tier 2 team for resolution.” Continuing our persistent efforts, JINC was finally listed on the Science Citation Index Expanded at the end of 2019, meaning that it would officially be granted its first impact factor in 2020 [3]. In addition to its first impact factor, the global COVID-19 pandemic dramatically increased the submissions of papers by more than four times relative to the previous year. This busy schedule provided an invaluable chance to broaden my professional perspectives.

The first decade of JINC’s history was indispensable for laying a solid foundation, but it was simply the past. Under the suitable perception and boundless contributions of intensivists and researchers worldwide, Professor Yoshifumi Kotake, the EIC, Deputy Editors, and Editorial Board members have actively and persistently made great efforts to advance JINC, magnify our specialty of medical science, and improve the quality of clinical practice, ultimately providing exceptional benefits to humanity.

I look forward to seeing the bright future of the JINC. A happy 10th anniversary!

## Data Availability

Not applicable.
